# Cold-perfusion decellularization of whole-organ porcine pancreas supports human fetal pancreatic cell attachment and expression of endocrine and exocrine markers

**DOI:** 10.1177/2041731417738145

**Published:** 2017-10-30

**Authors:** Erik Elebring, Vijay K Kuna, Niclas Kvarnström, Suchitra Sumitran-Holgersson

**Affiliations:** 1 Laboratory for Transplantation and Regenerative Medicine, Sahlgrenska Academy, University of Gothenburg, Gothenburg, Sweden; 2 The Transplant Institute, Sahlgrenska University Hospital, Gothenburg, Sweden

**Keywords:** Pancreas, decellularization, cold-perfusion, recellularization, human fetal pancreatic stem cells, insulin, glucagon, amylase

## Abstract

Despite progress in the field of decellularization and recellularization, the outcome for pancreas has not been adequate. This might be due to the challenging dual nature of pancreas with both endocrine and exocrine tissues. We aimed to develop a novel and efficient cold-perfusion method for decellularization of porcine pancreas and recellularize acellular scaffolds with human fetal pancreatic stem cells. Decellularization of whole porcine pancreas at 4°C with sodium deoxycholate, Triton X-100 and DNase efficiently removed cellular material, while preserving the extracellular matrix structure. Furthermore, recellularization of acellular pieces with human fetal pancreatic stem cells for 14 days showed attached and proliferating cells. Both endocrine (C-peptide and PDX1) and exocrine (glucagon and α-amylase) markers were expressed in recellularized tissues. Thus, cold-perfusion can successfully decellularize porcine pancreas, which when recellularized with human fetal pancreatic stem cells shows relevant endocrine and exocrine phenotypes. Decellularized pancreas is a promising biomaterial and might translate to clinical relevance for treatment of diabetes.

## Introduction

In 2014, 244 million adults worldwide were suffering from diabetes mellitus.^1^ From 1980 to 2014, the prevalence of diabetes among the world population has increased from 4.3% to 9.0% for men and from 5.0% to 7.9% for women. The increased levels of circulating blood glucose in patients suffering from diabetes can, over time, cause blindness, renal failure, or nerve damage.^2^ In 2012, 1.5 million patients died from causes directly related to diabetes, while the total deaths in the same year from high blood glucose levels were 3.7 million.^3^ Apart from the loss of lives, diabetes also causes economic burden. One study predicts diabetes will cause losses in gross domestic product around the world of US$1.7 trillion from 2011 to 2030.^4^

Traditionally, patients with diabetes mellitus have been treated with injections of exogenous insulin, anti-diabetic drugs, or behavioral changes. With these therapies, it is hard to keep insulin at therapeutic levels and hyperglycemia can occur as a result. Better long-term euglycemia can be achieved with whole pancreas or islet transplantation.^5^

Accelerated advances in the fields of tissue engineering and regenerative medicine can potentially increase the outcome of pancreas or islet transplantation. Today, islet transplantation and whole pancreas transplantation require high immunosuppression to prevent rejection, or alternatively an encapsulation that creates a barrier between the donated islets and recipient’s immune system.^6^ Major problems of current materials for encapsulation are limited biocompatibility, hypoxia, and delayed secretion of insulin. Most often, the materials used for encapsulation are non-human polymers that form hydrogels around the islets.

In the last decade, the technique of decellularization (DC) has emerged, where cells and cellular material are removed from a tissue while the extracellular matrix (ECM) scaffold is preserved.^7,8^ Along with the preservation of the ECM scaffold the idea of DC is also to maintain scaffold-bound growth factors and cytokines, which can be used for differentiation of progenitor cell into tissue-specific cells when the scaffold is recellularized. Since, in theory, the cell membrane associated antigenic epitopes are removed during the DC process, the probability of an acellular biomaterial to cause an immune response when allogenic or xenogeneic cells are introduced is less.

Previous studies of DC and recellularization (RC) of pancreas have been published.^9–13^ One study has also been published showing a proof of concept using acellular ECM scaffold as biomaterial for encapsulation of insulin-producing cells with improved glycemic control in diabetic mice.^14^

The field of DC and RC has evolved a lot over the past years, and the research has been focused toward kidney, liver, heart, and lungs. This has left tissue engineering of pancreas lagging behind despite the fact that more than 10% of global health expenditure is focused toward diabetes.^15^ The lack of research in tissue engineering of pancreas might be due to the challenging dual nature of pancreas. Pancreas consists of two distinct tissue types: endocrine (α-, β-, ε-, δ-, and PP-cells) and exocrine tissue (acinar and ductal cells).^16^ Glucagon secreted from α-cells and insulin secreted from β-cells keep blood glucose at a steady level. Acinar cells of pancreas secrete various digestive enzymes that together with bicarbonate secreted from ductal cells form the pancreatic juice that aids to digest food in duodenum.^17^ The digestive enzymes consist mainly of different proteases, but also lipases, nucleases, and amylases. The enzymes are produced and secreted in an inactive proenzyme form that requires active trypsin for an activation cascade of all proenzymes to start. During the process of DC, cells are lysed and intracellular proenzymes are exposed to the surroundings. It is likely that the interaction with the harsh DC environment can activate some of the proenzymes that in turn activate all other proenzymes in a chain reaction. Activated proteases will cause damage to the ECM scaffold, so these enzymes need to be inhibited for a controlled DC of pancreas. One way to achieve inhibition can be through regulating the temperature of the surroundings. It has been shown in rat that the hydrolysis efficiency decreases to about 20% at 5°C compared to temperature optimum, and it is a threefold decrease in hydrolysis efficiency from room temperature to 5°C.^18^ Previously, only one study has utilized cold temperature for pancreas DC, but neither discussing the need nor the effect.^11^ In addition to physical inhibition of enzymes at cold temperature, chemical serine protease inhibitors like phenylmethylsulfonyl fluoride (PMSF) can improve the quality of decellularized scaffold.^19^

This study aimed to introduce a novel approach for efficient perfusion DC of whole-organ porcine pancreas with inhibition of exocrine enzymes to maximize preservation of ECM ultrastructure. Acellular pancreas scaffolds were then repopulated with human fetal pancreatic stem cells (hFPSC) to demonstrate it as a suitable biomaterial for future tissue engineering of pancreas and islets.

## Materials and methods

### Whole-organ porcine pancreas isolation and harvest

Following local ethical guidelines, pancreata (n = 3) were harvested from euthanized, heparinized (400 IU/kg, LEO Pharma, Denmark) young normal pigs (30–50 kg) used for other studies or surgical training. To further ensure good heparinization, cold phosphate-buffered saline (PBS, pH 7.4) with heparin (17 IU/mL) was administrated through portal vein until whole pancreas felt cold. Intact pancreata with all three lobes (connective, duodenal, and splenic lobe) were excised together with duodenal segment stretching from duodenal to connective lobe. Both the aorta and the portal vein were harvested along with each pancreas. During backbench work, one side of the aorta was cannulated, while all other vasculature outlets except the portal vein were ligated. Organs were flushed with 5 mM disodium ethylenediaminetetraacetic acid (EDTA, Alfa Aesar, Germany) and 0.02% (w/v) sodium azide (Sigma-Aldrich, USA) in cold (4°C) ultrapure water for 1 h at 1.2 L/h speed using peristaltic pump (Oina, Sweden) in series with degasser (Biotech, Sweden) to remove blood. Flushed pancreata were frozen at −20°C in PBS with 5 mM EDTA and 0.02% sodium azide until start of DC.

### Cold-perfusion DC

The frozen pancreas was gradually thawed and connected to perfusion system (peristaltic pump and degasser connected in series) at 4°C and perfusion of 1.2 L/h at 4°C started. To further wash out blood remnants, the organ was perfused with distilled water containing 5 mM EDTA and 0.02% (w/v) sodium azide overnight. Briefly, cold-perfusion DC was achieved by consecutive flow-through perfusion of 4% (w/v) sodium deoxycholate (SDC, Sigma-Aldrich, Italy) combined with 6% (v/v) Triton X-100 (Alfa Aesar, Germany) and 0.4 mM PMSF (Roche, Germany) for 8 h followed by washing with distilled water for 96 h, 40 IU/mL deoxyribonuclease I (DNase I, Worthington, USA) in Dulbecco’s PBS with calcium chloride and magnesium chloride (Sigma-Aldrich, UK) for 4 h at 37°C, and finally, 120 h of wash with distilled water. All DC solutions contained 0.02% (w/v) sodium azide to prevent bacterial growth. Decellularized pancreata were kept in PBS with 0.02% (w/v) sodium azide, at 4°C for short time or at −20°C for long-time storage.

### Culture of hFPSC

Cells isolated from pancreas of legally aborted human fetus as described by us earlier were expanded.^20^ The isolation protocol was approved by the local ethics committee. The cells were cultured in human placental collagen-coated culture flasks with pancreas differentiation medium (Dulbecco’s Modified Eagle Medium with high glucose (Lonza, Belgium), 1% l-glutamine (Gibco, UK), 1% antimycotic-antibiotic (Gibco, USA), 10% heat-inactivated human AB serum (Sigma-Aldrich, USA), 50 μM ethanolamine (Sigma-Aldrich, USA), 50 μM O-phosphoethanolamine (Sigma-Aldrich, USA), 0.1 nM liothyronine (Sigma-Aldrich, USA), 20 ng/mL recombinant human IGF-1 (PeproTech, USA), and 10 ng/mL human recombinant prolactin (Peprotech, USA)).

### Recellularization of cold-perfusion decellularized porcine pancreas scaffolds

Approximately 1 cm^2^ pieces of cold-perfusion decellularized porcine pancreas scaffolds were cut perpendicular to splenic lobe prolongation for preparation for RC. Pieces were sterilized in 0.1% peracetic acid (Sigma-Aldrich, Germany) in PBS for 70 min at 37°C and washed repeatedly in PBS with 1% antimycotic-antibiotic (Gibco, USA). Scaffolds were placed in 12-well Transwell^®^ permeable supports (0.4 μm pore size, Costar, USA), and 0.5 million hFPSC (at passage 6) were seeded onto each scaffold. Scaffolds were incubated at 37°C for 1 h after cell seeding followed by addition of pancreas differentiation media. Tissue pieces were harvested for characterization at day 5 (n = 3) and day 14 (n = 3).

### Histology, immunohistochemistry, and immunofluorescence

Tissue biopsies (normal, DC and RC) were processed according to standard procedure. Biopsies were fixed in 4% phosphate-buffered formaldehyde (Histolab, Sweden) for 24 h and dehydrated with ascending concentrations of ethanol (Histolab, Sweden) and X-TRA-Solv (Medite, Germany) before embedding in paraffin (Histolab, Sweden). Samples were cut in 5 μm thickness cross sections using a microtome (Thermo Fisher Scientific, USA).

To analyze general histology of all biopsies, sections were stained with hematoxylin & eosin (H&E, Histolab, Sweden) and 4′,6-diamidino-2-phenylindole (DAPI, Life Technologies, USA) according to standard procedures. H&E staining of sections from porcine pancreata (n = 2) decellularized with a protocol similar to the one used in this study, but at room temperature was also performed for comparison of ECM structure. Biopsies from normal and decellularized tissue were stained with Masson’s trichrome (MT, Polysciences, USA) and Verhoeff van Gieson (VVG, Polysciences, USA) according to manufacturer’s instructions.

Immunohistochemical analysis of sections from recellularized scaffold was done with primary antibodies specific to pancreatic α-amylase (1:1000, ab21156, Abcam, UK). Normal human pancreas (Abcam, UK) was included as reference. For identification of specific staining horseradish peroxidase–conjugated broad-spectrum secondary antibody (Invitrogen, USA) was used. Briefly, sections were rehydrated, antigen retrieved, endogenous peroxidases blocked, and protein blocked before incubating with primary antibodies overnight at 4°C. Slides were washed and incubated with secondary antibody for 10 min at room temperature. Colorimetric identification was performed using 3,3′-diaminobenzidine (DAB, Dako, USA) and counter-stained with hematoxylin before dehydration and then cover-slipped. Sections with only secondary antibody were included as negative control.

To further analyze function of cells in recellularized scaffolds, immunofluorescent stainings were performed with antibodies against C-peptide (1:500, ab8297, Abcam, UK), pancreatic and duodenal homeobox 1 (PDX1, 1:150, ab84987, Abcam, UK), glucagon (1:750, ab10988, Abcam, UK), and proliferating cellular nuclear antigen (PCNA, 1:1000, ab184660, Abcam, UK). Normal human pancreas (Abcam, UK) and human fetal kidney were included as reference. Alexa Fluoro 568 conjugated immunoglobulin G (IgG) goat anti-mouse secondary antibody (A11031, Thermo Fisher Scientific, USA) was used. Briefly, sections were rehydrated, antigen retrieved, and blocked with 5% goat serum before incubating with primary antibodies overnight at 4°C. Slides were washed and incubated with secondary antibody for 45 min at room temperature and then counter-stained with DAPI before cover-slipping. Sections with only secondary antibody were included as negative control. Unspecific background staining was reduced digitally in software (Leica, Germany).

### DNA and ECM protein quantifications

For DNA quantification, biopsies were taken from splenic lobe of normal (n = 3) and decellularized (n = 3) pancreata and after 5 and 14 days of RC (n = 3, respectively). Normal biopsies were taken from newly euthanized pigs and biopsies were placed in liquid nitrogen immediately for storage until use. Before the start of DNA extraction, biopsies were placed on wipes to remove excess fluid and weighed. DNA was extracted using GenElute™ Mammalian Genomic DNA Miniprep Kit (Sigma-Aldrich, USA) according to manufacturer’s instructions (RNase step included). Isolated double-stranded DNA was quantified using Qubit^®^ dsDNA HS Assay Kit (Thermo Fisher Scientific, USA).

ECM components were quantified in duplicate biopsies of splenic lobe from normal (n = 3) and decellularized (n = 3) pancreata. Collagen (soluble and insoluble), elastin, and glycosaminoglycans (GAGs) were quantified with Sircol, Fastin, and Blyscan assays (Biocolor, UK), respectively. All assays were performed according to manufacturer’s instructions.

### Qualitative analysis of damaging effect from pancreatic extract

To further understand the damaging effect of pancreatic exocrine enzymes on cells and tissue, normal porcine pancreas tissue was homogenized in ice-cold PBS and then centrifuged at 2000 r/min at 4°C for 20 min. Supernatant was collected, re-centrifuged at 4000 r/min at 4°C for 20 min, and lyophilized. Porcine kidney isolate extracted with same procedure was included as a low-enzymatic activity control to exclude xenogeneic effects. The protein content in each powder was measured with standard Bradford method (Bio-Rad, USA). Isolates were resuspended in standard cell culture media (1 mg protein/mL media) and sterile filtered. Prepared cell culture media (with pancreas and kidney powder, n = 2, respectively) were added to cultures of human mesenchymal stem cells (MSCs) in 6-well format along with negative (only media) and positive (0.05% trypsin, Gibco, UK and 1% sodium dodecyl sulfate (SDS), Sigma-Aldrich, USA, respectively) controls. Photos were taken before addition and at 10 min 1 h and 4 h time points.

### Statistics

All values and graphs represent group mean, while error bars represent the standard error of the mean. The graphs were plotted using GraphPad Prism 7.0. Welch’s t-test was used to analyze significant differences between groups after normality for each group had been confirmed with Shapiro–Wilk test. A p < 0.05 was considered significant.

## Results

### Morphology after cold-perfusion DC

**Figure 1.**
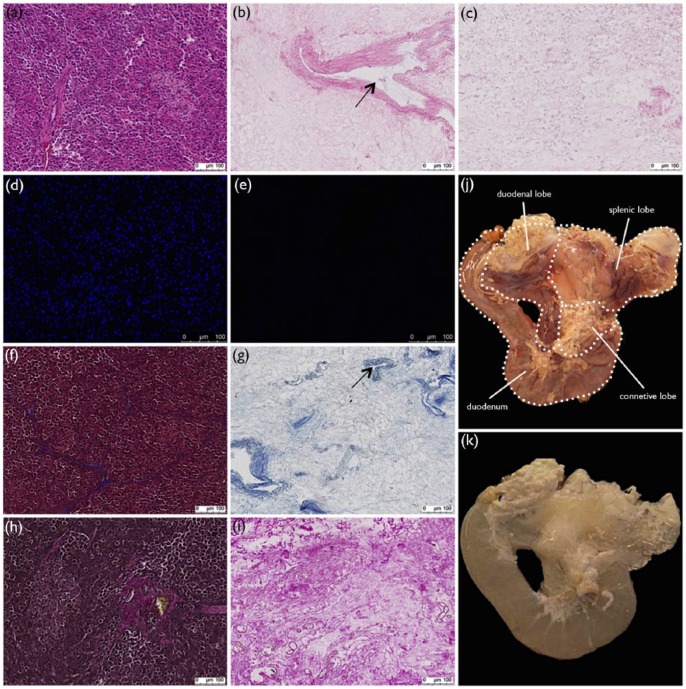
Histological and morphological evaluation of cold-perfusion decellularization. Histological comparison of normal and cold-perfusion decellularized porcine pancreas by (a) and (b) H&E, (d) and (e) DAPI, (f) and (g) MT, and (h) and (i) VVG stainings showing complete nuclei and cytoplasm removal while preserving ECM and vasculature (arrows). With H&E stainings of (b) cold-perfusion and (c) room-temperature decellularized porcine pancreata, it could be concluded that cold-perfusion preserved ECM structure more. Small circular structures, believed to be cytoplasmic remnants, could be seen in pancreata decellularized in room temperature. Gross evaluation revealed a distinct change in appearance (j) before and (k) after decellularization. Graphic representation of duodenum, connective lobe, duodenal lobe, and splenic lobe can be seen.

Intact decellularized porcine pancreas was obtained by perfusion of 4% SDC and 6% Triton X-100 at 4°C followed by DNase I treatment. A distinct color change could be observed as a result of DC. Washed normal porcine pancreas has a pinkish color (Figure 1(j)), whereas after DC, pancreas was totally white in appearance (Figure 1(k)). The color change of duodenum indicates an extensive vascular connection between pancreas and intestine. During DC process, a complete perfusion could be seen with aorta as inlet and portal vein as outlet.

### Characterization of decellularized porcine pancreas scaffolds

H&E staining of cold-decellularized porcine pancreas (Figure 1(b)) showed absence of nuclei as compared to normal porcine pancreas (Figure 1(a)), where purple-stained nuclei were seen. H&E staining of porcine pancreas decellularized in cold environment also showed a more intact ECM structure as compared to H&E staining of porcine pancreas decellularized with a comparable protocol at room temperature (Figure 1(c)). Pancreas decellularized at room temperature had a more ruptured ECM structure and staining patterns reminiscent of cytoplasmic residues. DAPI staining of normal (Figure 1(d)) and DC porcine pancreas (Figure 1(e)) confirmed complete washout of intact nuclei. MT stainings (Figure 1(f) and (g)) showed absence of cytoplasm (red) after DC, while collagen fibers (blue) were retained. Retention of collagen fibers (pink) was confirmed with VVG stainings (Figure 1(h) and (i)). Histological stainings of DC tissue also confirmed preservation of vasculature structures (arrows, Figure 1(b) and (g)).

**Figure 2.**
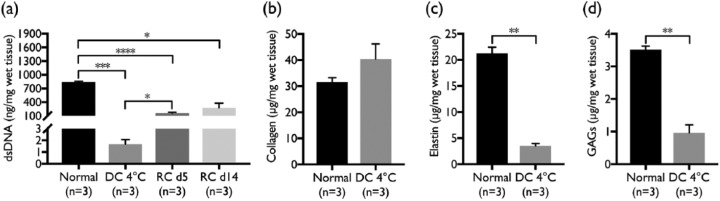
DNA and ECM quantification. Quantification of (a) double-stranded DNA (dsDNA) demonstrated a significant (p = 0.0005) decrease after decellularization compared to normal porcine pancreas and a significant (p = 0.0137) increase after 5 days of recellularization when compared to cold-perfusion decellularized tissue. Recellularized tissue harvested after 5 and 14 days showed significantly (p < 0.0001 and p = 0.0264) lower DNA content compared to normal porcine pancreas. Quantification of ECM proteins (b) collagen (soluble and insoluble combined), (c) elastin, and (d) GAGs showed no significant change of collagen, while both elastin and GAGs decreased significantly (p = 0.0021 and 0.0034, respectively).

DNA quantification of biopsies from normal and DC tissues showed a significant (p = 0.0005) decrease in the double-stranded DNA with DC (Figure 2(a)). Double-stranded DNA decreased from 840 ± 18 to 1.67 ± 0.4 ng/mg with DC.

ECM quantification demonstrated no significant change in collagen (soluble and insoluble combined) content (Figure 2(b)) after DC, while elastin (p = 0.0021, Figure 2(c)) and GAGs (p = 0.0034, Figure 2(d)) decreased significantly. The collagen, elastin, and GAGs contents in decellularized pancreas were 40.4 ± 5.8, 3.54 ± 0.42 and 0.96 ± 0.25 µg/mg, respectively, as compared to 31.5 ± 1.7, 21.2 ± 1.2 and 3.5 ± 0.11 µg/mg for normal porcine pancreas, respectively.

### Harmful effect of pancreatic extract

**Figure 3.**
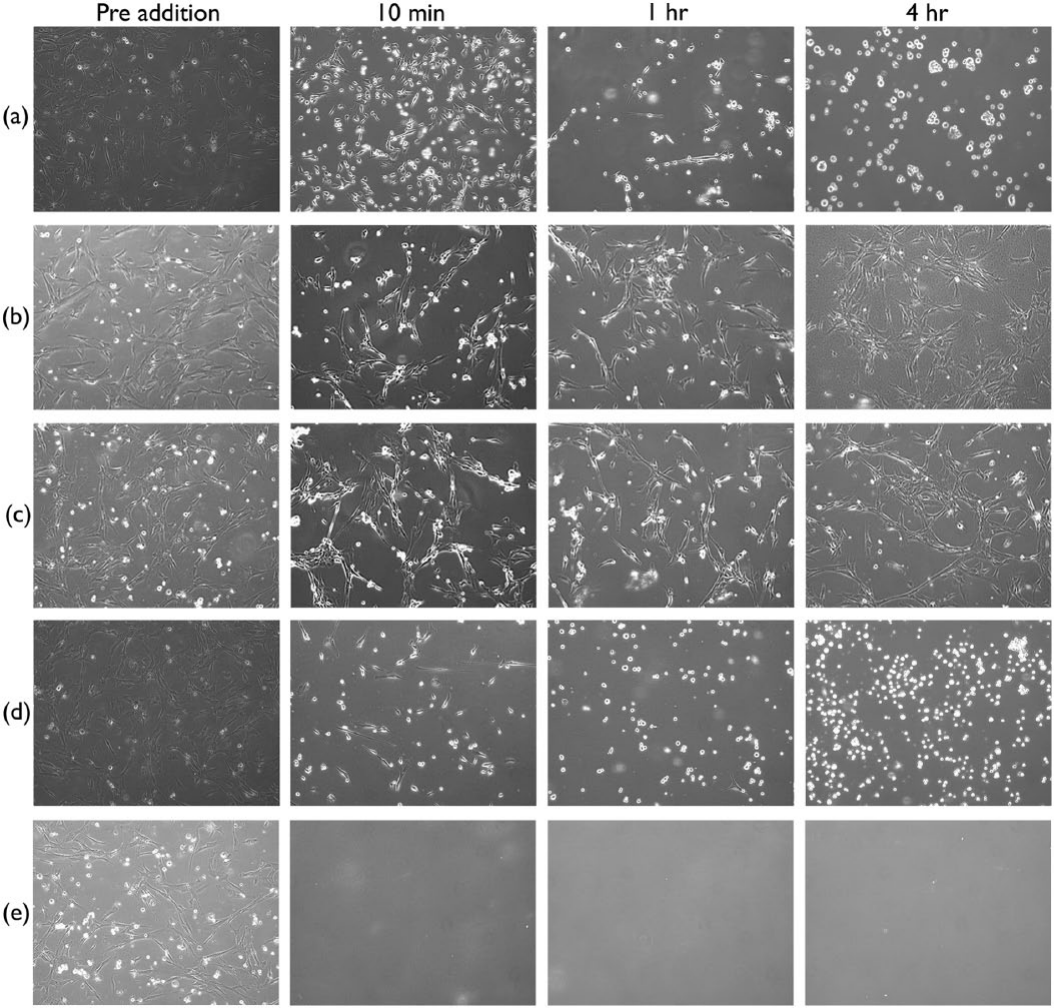
Damaging effect of pancreatic isolate. Survival of mesenchymal stem cells after addition of pancreas isolate was studied qualitatively at 10 min, 1 h and 4 h after addition. Addition of (a) porcine pancreas isolate had an acute effect more comparable to (d) trypsin than (e) SDS treatment, where cell disintegration could be seen already after 10 min. With addition of (b) porcine kidney isolate, no effect could be seen even after 4 h. Also, for (c) negative control, no effect could be seen after 4 h.

Isolated porcine pancreatic extract showed a clear cytotoxic effect (Figure 3(a)) on two-dimensional (2D) culture of human MSCs, more comparable to the addition of trypsin (Figure 3(d)) than SDS (Figure 3(e)). The effect was acute already after 10 min and almost all cells detached from the cell culture plate. At 4 h, a visible difference in cell numbers could be noted between wells treated with pancreatic extract and wells treated with trypsin. More cells were seen in trypsin control. In controls treated with SDS, a complete cell disintegration could be seen already after 10 min. Similar isolate from porcine kidney tissue showed no or minimal effect (Figure 3(b)) on cultured cells, akin to negative control (Figure 3(c)).

### Recellularization of cold-perfusion decellularized scaffolds with hFPSC

**Figure 4.**
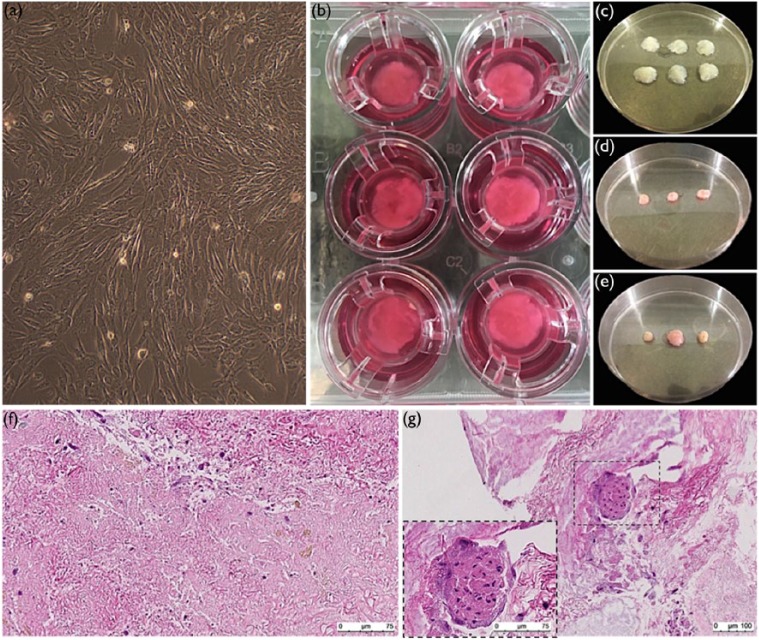
Recellularization of acellular pancreas scaffolds with human fetal pancreatic stem cells. Human fetal pancreatic stem cells were (a) expanded and (b) seeded onto acellular pancreas scaffolds and cultured in permeable supports. A slight increase in the tissue density could be seen when comparing scaffolds (c) before and scaffolds harvested at (d) day 5 and (e) day 14. H&E stainings of harvested scaffolds showed areas where cells were (f) well-spread (purple nuclei) and other areas where no cells could be seen. In some of harvested scaffolds (g) cluster-like aggregations of cells could be seen.

Expanded hFPSC (Figure 4(a)) were seeded onto sterilized pieces of cold-perfusion decellularized porcine pancreas scaffold (Figure 4(c)). Scaffolds were cultured in Transwell^®^ permeable supports (Figure 4(b)), and pieces were harvested at day 5 (Figure 4(d)) and day 14 (Figure 4(e)). Shrinkage in tissue volume could be seen with RC (compare Figure 4(c) with Figure 4(d) and (e)). H&E staining of recellularized scaffolds revealed presence of cells (purple nuclei in Figure 4(f) and (g)). For none of the pieces, complete cell coverage was seen. Instead, the coverage of cells varied within each piece; in some areas, cells were well-spread (Figure 4(f)), but in other areas, no cells could be seen. In some of the pieces, cells had aggregated together into cluster-like structures (arrow, Figure 4(g)). No significant difference regarding cell numbers and coverage could be seen between scaffolds harvested at day 5 and day 14.

### Characterization of recellularized pancreatic scaffolds

Contents of double-stranded DNA in recellularized scaffolds harvested at day 5 and day 14 were 159 ± 32 and 278 ± 171 ng/mg, respectively (Figure 2(a)). A significant (p = 0.0137) increase in the double-stranded DNA could be seen when comparing acellular tissue and recellularized tissue harvested after 5 days, but not for 14 days of RC. However, as expected DNA contents of recellularized samples were lower than for normal porcine pancreas (p < 0.0001 and p = 0.0264, respectively).

**Figure 5.**
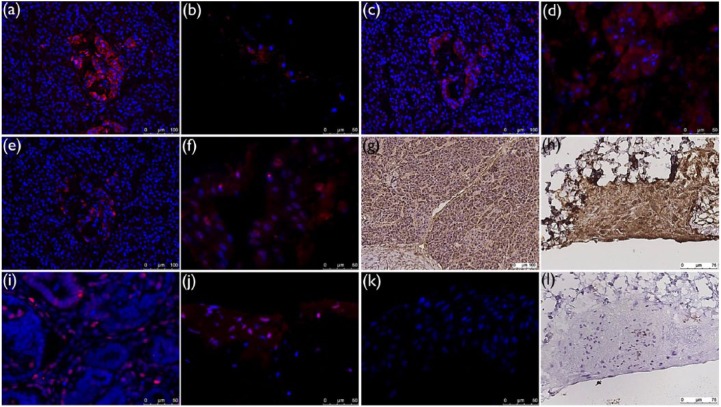
Endocrine and exocrine markers expression in recellularized scaffolds. Immunostainings showed expression of (b) C-peptide (red), (d) PDX1 (red), (f) glucagon (red), and (h) α-amylase (brown) in recellularized scaffolds. Recellularized scaffolds also expressed (j) PCNA (red) in nuclei in fraction of cells. No significant difference between scaffolds harvested at day 5 and day 14 was seen. Positive control stainings could be seen for (a) C-peptide (red), (c) PDX1 (red), (e) glucagon (red), and (g) α-amylase (brown) in normal human pancreas and for (i) PCNA (red) in human fetal kidney. No or less background stainings were seen in (k) immunofluorescence and (l) immunohistochemistry stainings of recellularized scaffolds without primary antibodies.

Immunological stainings for pancreas function showed both endocrine and exocrine tissue properties. Immunofluorescence stainings revealed both C-peptide (red, Figure 5(b)), that is, insulin and glucagon (red, Figure 5(f)) secretions as well as transcription factor PDX1 expression (red, Figure 5(d)) in all recellularized scaffolds. Also exocrine function through α-amylase (brown, Figure 5(h)) secretion was seen in all recellularized scaffolds. No significant difference regarding endocrine or exocrine function between scaffolds harvested at day 5 or day 14 was seen. Expression of PCNA (red, Figure 5(j)) in nuclei was seen in fraction of cells, indicating proliferative capacity for these cells. No significant difference in PCNA expression could be seen between day 14 and day 5. Staining of normal human pancreas as positive control for pancreas markers C-peptide, PDX1, glucagon, and α-amylase (Figure 5(a), (c), (e), and (g), respectively) and human fetal kidney as positive control for PCNA (Figure 5(i)) confirmed specificity of primary antibodies. Negative controls (recellularized scaffolds without primary antibodies, Figure 5(k) and (l)) showed no or weak background staining.

## Discussion

In this study, we present a cold-perfusion protocol that successfully decellularizes whole porcine pancreas, which in turn supports endocrine function of hFPSC. Cold-perfusion DC of pancreas has previously been utilized in a study by Peloso et al.,^11^ but without discussing the reason any further. The protocol for cold-perfusion DC used in this study significantly reduces the detergent exposure time compared to previous attempt.^11^ Here, we also present evidence for the damaging effect of exocrine enzymes released from acinar cell lysis during DC. When porcine pancreas isolate was introduced into cell culture of human MSCs, the damaging effect was acute and more comparable to the trypsin treatment than SDS treatment. In addition, less cells were seen after 4 h of treatment with porcine pancreas isolate as compared to treatment with trypsin. This might indicate other effects of pancreas isolate than trypsinization. For example, this might indicate cell membrane damaging effects of pancreatic lipases resulting in cell disintegration similar to SDS treatment. No damaging effect was seen with kidney isolate, indicating the effect is pancreas-specific and not due to xenogeneic effect. Released exocrine enzymes can theoretically be beneficial during DC since they facilitate both cell lysis and breakage of ECM-cell interactions. However, since the release of exocrine enzymes during DC of pancreas is uncontrolled, they must be inhibited to achieve a stable and reproducible DC protocol and to prevent exocrine enzymes from damaging ECM structures. It is known that both physical and chemical inhibitors can be used to decrease exocrine enzyme activity.^18,19^ Therefore, we hypothesized that DC at cold temperature in combination with the serine protease inhibitor PMSF and continuous perfusion washout of released exocrine enzymes could facilitate complete DC while keeping ECM structures intact. In the very beginning of the iterative process of developing a DC protocol for porcine pancreas, we spent a considerable amount of time on room-temperature protocols before realizing cold-perfusion protocols might be superior. When comparing histological stainings of the cold-perfusion protocol to a similar DC protocol performed at room temperature, both protocols resulted in complete nuclear washout. With cold-perfusion DC, a more intact ECM structure was achieved with long-spanning fibers, while when decellularizing pancreas at room temperature, leftover ECM was more ruptured.

Histological stainings showed an intact ECM structure after cold-perfusion DC. Importantly, preserved vascular structures could also be seen post DC. The extensive vascular connection between duodenum and pancreas was visualized by the color change of duodenal segment with DC of pancreas. In order to accomplish complete perfusion, all these vascular connections have to be ligated or more convenient, as presented in this study, the pancreas is dissected along with the upper duodenal segment. Preserved vasculature after DC together with dissection strategy to keep the upper duodenal segment intact makes it a suitable starting platform for whole-organ RC.

Cold-perfusion DC of porcine pancreas preserves the collagen content in the scaffold. Collagen fibers not only provide physical strength to the tissue but also are required for cell adhesion, migration, and differentiation.^21^ The significant decrease in the GAGs is a drawback with the protocol, since GAGs bind and store growth factors in the ECM.^22^ The depletion of tissue-specific growth factors may influence cell fate during RC.

Despite the depletion of GAGs, the successful RC of cold-perfusion decellularized porcine pancreas scaffolds with hFPSC proves it to be a non-toxic scaffold suitable for cell seeding. Shrinkage in tissue volume could be an indication of cell adhesion bringing tissue together. Cell coverage after RC was not complete, and a variation within each recellularized scaffold could be seen. Cell coverage could probably be improved by increasing seeding density or by seeding cells through the vasculature. The hFPSC used in this study could be advantageous over other stem cell sources to obtain pancreatic function due to the committed pancreatic lineage of these cells.

Successful RC seen with histology stainings was confirmed with quantification of double-stranded DNA in recellularized tissue. A significant increase in the DNA content was seen after 5 days of RC compared to acellular scaffold, even though this significant difference was not seen after 14 days of RC. It may be possible that with a larger sample size the increase would also have been significant at 14 days of RC.

Even though cell coverage was not complete, the recellularized scaffolds showed both endocrine and exocrine properties after RC with hFPSC. Endocrine properties could be seen through synthesis of both C-peptide and glucagon. C-peptide indicates insulin production since it is a by-product of the conversion of proinsulin to functional insulin.^23^ Along with endocrine function, expression of transcription factor PDX1 could be seen. This transcription factor is central in β-cell function and survival. Even though PDX1 is a transcription factor that is normally localized to the nucleus, it has previously been shown that insulin induces re-localization of PDX1 from nucleus to cytoplasm.^24^ In this study PDX1 was observed in the cytoplasm of cells, which further indicates insulin function in recellularized scaffolds. Furthermore, α-amylase secretion, a feature of exocrine tissue, was also detected.

After whole pancreas transplantation, graft rejections are known to occur due to exocrine pancreatitis, and therefore, it may be postulated that such patients might benefit from transplantation of only endocrine tissue.^25^ However, during pancreas development, it has been suggested that the exocrine tissue has a clear impact on the physiology of islet and endocrine tissue.^26^ Growth factors and cytokines secreted from exocrine cells have been shown to be involved in islet differentiation. Therefore, in this study, hFPSC were used since we believed that the heterogeneous population of these progenitor cells would give rise to both endocrine and exocrine tissues. Furthermore, these cells have high proliferative capacity with committed lineage to develop into pancreas. With this strategy, islet differentiation might benefit from simultaneous development of exocrine tissue and secretions of growth factors and cytokines from the exocrine tissue that triggers endocrine development. Fetal stem cells have been used to treat various diseases since 1928, but still ethical issues have not been completely resolved and a shift toward other sources of progenitor cells, that is, induced pluripotent stem cells, is impending.^27^

Expression of proliferation marker PCNA in recellularized scaffolds harvested at both day 5 and day 14 indicates DNA is still being replicated in these cells and proliferation is occurring. Thus, proliferating cells at day 14 demonstrate that scaffolds of decellularized pancreas are suitable for long-time culture of cells.

## Conclusion

To conclude, this study demonstrates cold-perfusion as an efficient method for DC of whole porcine pancreas. Characterization of decellularized pancreas revealed satisfactory removal of cells while preserving important vascular structures and ECM components. This study also clearly shows the damaging effect of pancreas isolate on cells cultured, which further motivates that inhibition of exocrine enzymes during DC of pancreas is important. In addition, initial RC experiments proved cold-perfusion decellularized porcine pancreas scaffolds were non-toxic, and when recellularized with hFPSC, it can support growth and proliferation of both endocrine and exocrine cells.
